# Variations in the management of canine osteoarthritis: a cross-sectional survey of veterinary practices

**DOI:** 10.3389/fvets.2026.1814641

**Published:** 2026-05-29

**Authors:** Elyza Bird, Erin Miscioscia, Christina Montalbano, James Colee, Jennifer Repac

**Affiliations:** 1 College of Veterinary Medicine, University of Florida, Gainesville, FL, United States; 2 Institute of Food and Agricultural Sciences Statistical Consulting Unit, University of Florida, Gainesville, FL, United States

**Keywords:** ACVSMR, bedinvetmab, canine, grapiprant, librela, multimodal, osteoarthritis, supplements

## Abstract

**Introduction:**

There is limited data on how veterinarians implement multimodal therapies for managing canine osteoarthritis (OA). The primary aim of this study was to describe how OA is managed by veterinarians with diverse backgrounds. The secondary aim was to report perceived side effects associated with bedinvetmab (Librela^TM^) and grapiprant. The authors hypothesized that variation exists in management patterns among veterinarians with different training backgrounds and experience levels.

**Methods:**

A cross-sectional, observational, voluntary response survey was distributed to veterinarians assessing respondent demographics, diagnostic approaches, the frequency of treatment use for canine OA, and perceived adverse events associated with grapiprant and bedinvetmab.

**Results:**

373 responses were analyzed. Non-steroidal anti-inflammatory drugs (NSAIDs) were the most prescribed drug class, and gabapentin, bedinvetmab, and polysulfated glycosaminoglycan (Adequan^®^) were the most prescribed non-NSAID medications. The most used nutraceutical and non-pharmacologic treatments were omega-3 fatty acids and weight management. Fifty-three percent of respondents used imaging whenever possible to confirm OA. Those with higher levels of training (specialty training, rehabilitation certificates) and more years of experience used imaging more often. American College of Veterinary Surgery (ACVS) diplomates were less likely to use bedinvetmab and cannabainoids (CBD). American College of Veterinary Sports Medicine and Rehabilitation (ACVSMR) diplomates and rehabilitation-certified veterinarians were less likely to use glucosamine/chondroitin and bedinvetmab and more likely to use ketamine, amantadine, polysulfated glycosaminoglycan, green-lipped mussel, CBD, undenatured type II collagen, eggshell membrane, curcumin, and Boswellia serrata. Diplomates, rehabilitation-certified veterinarians, and more experienced veterinarians used non-pharmacological treatments, including intra-articular injections more often. The most reported perceived side effects of bedinvetmab were paresis/ataxia/proprioceptive deficits (31%) and polyuria/polydipsia (25%). Diarrhea (33%), and vomiting (31%) were the most reported perceived side effects of grapiprant.

**Conclusion:**

There is variation in multimodal approaches to canine OA management influenced by both training and years of clinical experience. While NSAIDs, omega-3 fatty acids, and weight management remain foundational, this study demonstrates a spectrum of utilized adjunctive strategies. The differences highlight opportunities for further research into the safety and efficacy of commonly used therapies. Reported perceived adverse events associated with bedinvetmab and grapiprant underscore the need for pharmacovigilance and further research investigating safety.

## Introduction

Canine osteoarthritis (OA) is a chronic, progressive, and degenerative disease affecting articular joints, which includes articular cartilage deterioration, bone remodeling with osteophyte formation, periarticular tissue damage, and low-grade inflammation (1, 2). Canine OA is highly prevalent, with reports ranging from 2.5% to over 35% (3–5). Despite its prevalence, early clinical signs of OA often go unnoticed, complicating timely diagnosis. The consequences of OA extend well beyond joint degeneration, leading to chronic pain, reduced mobility, and diminished quality of life, often manifesting as behavioral changes (1). Effective management of OA is essential for both preserving animal comfort and reducing the emotional burden experienced by pet owners. An association has been found between OA-related pain and impairment, caregiver burden, and owner consideration of euthanasia (6).

The management of canine OA is multimodal, often combining pharmaceutical, nutraceutical, nutritional, rehabilitative, and surgical interventions to target pain and inflammation (1). Nonsteroidal anti-inflammatory drugs (NSAIDs) and anti-nerve growth factor monoclonal antibody (bedinvetmab, Librela^TM^) are proposed as first-line agents (7, 8). Adjunct pharmaceutical options include gabapentin, amantadine, ketamine, tramadol, acetaminophen, tricyclic antidepressants, muscle relaxants, locoregional anesthetics, and opioids (8). Nutraceutical options include marine-sourced omega-3 fatty acids, undenatured type II collagen, glucosamine and chondroitin, cannabidiol products, green-lipped mussel, eggshell membrane, curcumin, and Boswellia serrata, among others (7–11). Non-pharmacologic strategies, such as weight management, hydrotherapy, low-level laser therapy (photobiomodulation), therapeutic ultrasound, pulsed electromagnetic field therapy, extracorporeal shockwave therapy (ESWT), electrotherapy, and acupuncture, have also been used to treat canine OA (1, 8, 12–14). Intra-articular (IA) injections and regenerative biologics like platelet-rich plasma and stem cells are increasingly utilized (1, 7, 8) as well as intramuscular polysulfated glycosaminoglycan (PSGAG, Adequan^®^) injections (15).

Given the array of OA management options, it is essential to evaluate how veterinarians diagnose and treat the disease. The primary aim of this study was to describe how osteoarthritis is managed across a diverse group of veterinarians. The secondary aim was to report perceived side effects associated with newer OA medications, specifically bedinvetmab and grapiprant. The authors hypothesized that variation exists in management patterns among veterinarians with different training backgrounds and experience levels.

## Materials and methods

### Survey design

An observational, cross-sectional, voluntary response study was conducted among veterinarians within the United States and international veterinarians with Doctor of Veterinary Medicine (DVM) equivalent degrees. A 16-question online survey (Supplementary File 1) was developed using the secure web-based platform Qualtrics (Qualtrics, Provo, UT). The survey questions requested responses regarding each veterinarian's years in practice and level of specialization, then explored canine OA management patterns including diagnostic modalities, pharmaceutical choices, nutraceutical/supplement use, and adjunctive therapies such as physical rehabilitation. Multiple choice (9 questions), multiple answer (4 questions), and Likert scale (3 questions) questions were included. Multiple answer questions allowed each respondent to select all answer choices that applied, therefore results for those questions are not mutually exclusive. Likert scale questions utilized the following scale: never = 1, rarely = 2, sometimes = 3, often = 4, always = 5. Conditional branching sent respondents to follow-up questions based on prior answers. The questionnaire was provided to 3 practitioners prior to general distribution to ensure that it was well understood and to determine the time required to complete the survey (approximately 5 min).

### Data collection

Participation in the study was optional and anonymous, and the only requirement for study participation was that the respondents be veterinarians within the United States or international veterinarians with DVM-equivalent degrees. The survey was distributed with an introductory page explaining project goals in order to reach the appropriate respondent population. The survey was distributed electronically via professional veterinary organizations, online veterinary communities, veterinary social media groups, and conference-related mailing lists to encourage broad participation from both general practitioners and veterinary specialists and was open to responses from January 1 through April 21, 2025. The project qualified for exemption from the University of Florida's Institutional Review Board (IRB) because it involved anonymous, voluntary survey data with no collection of identifiable information and no animal or client intervention.

### Statistical analysis

Three statistical approaches were employed according to the nature of the data: categorical versus categorical associations were examined with Pearson's chi-square test; when a categorical variable was compared to a Likert type response, the Wilcoxon rank-sum test was applied; and associations between two Likert type scores were evaluated using Spearman's rank correlation. The figures in this manuscript present individual-data points and percentages; the text reports only overall proportions and the corresponding test statistics where relevant. A two tailed *p*-value of < 0.05 was considered significant. All analyses were performed in JMP 18.

## Results

### Respondent demographics

**Figure 1:**
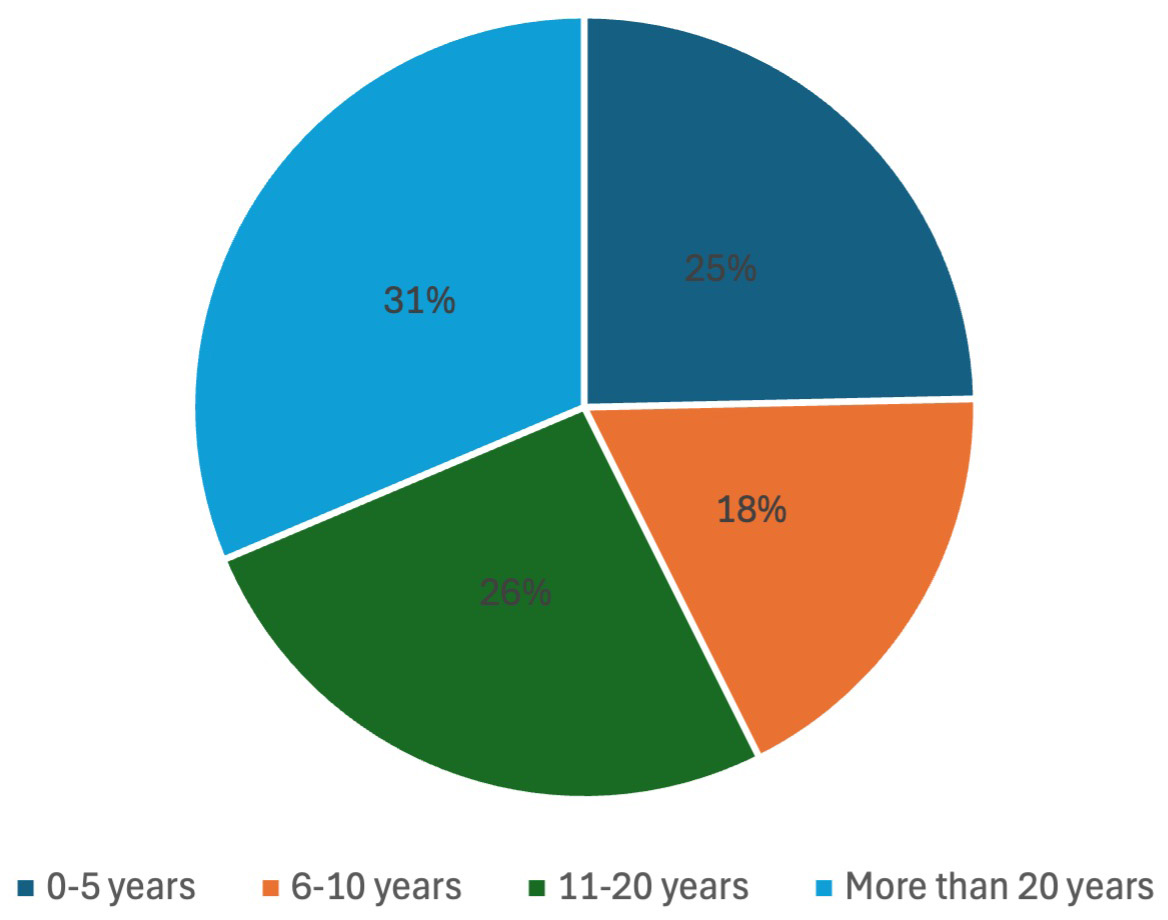
Distribution of respondents based on years in clinical practice. 0–5 years = dark blue; 6–10 years = orange; 11–20 years = green; more than 20 years = light blue.

**Figure 2:**
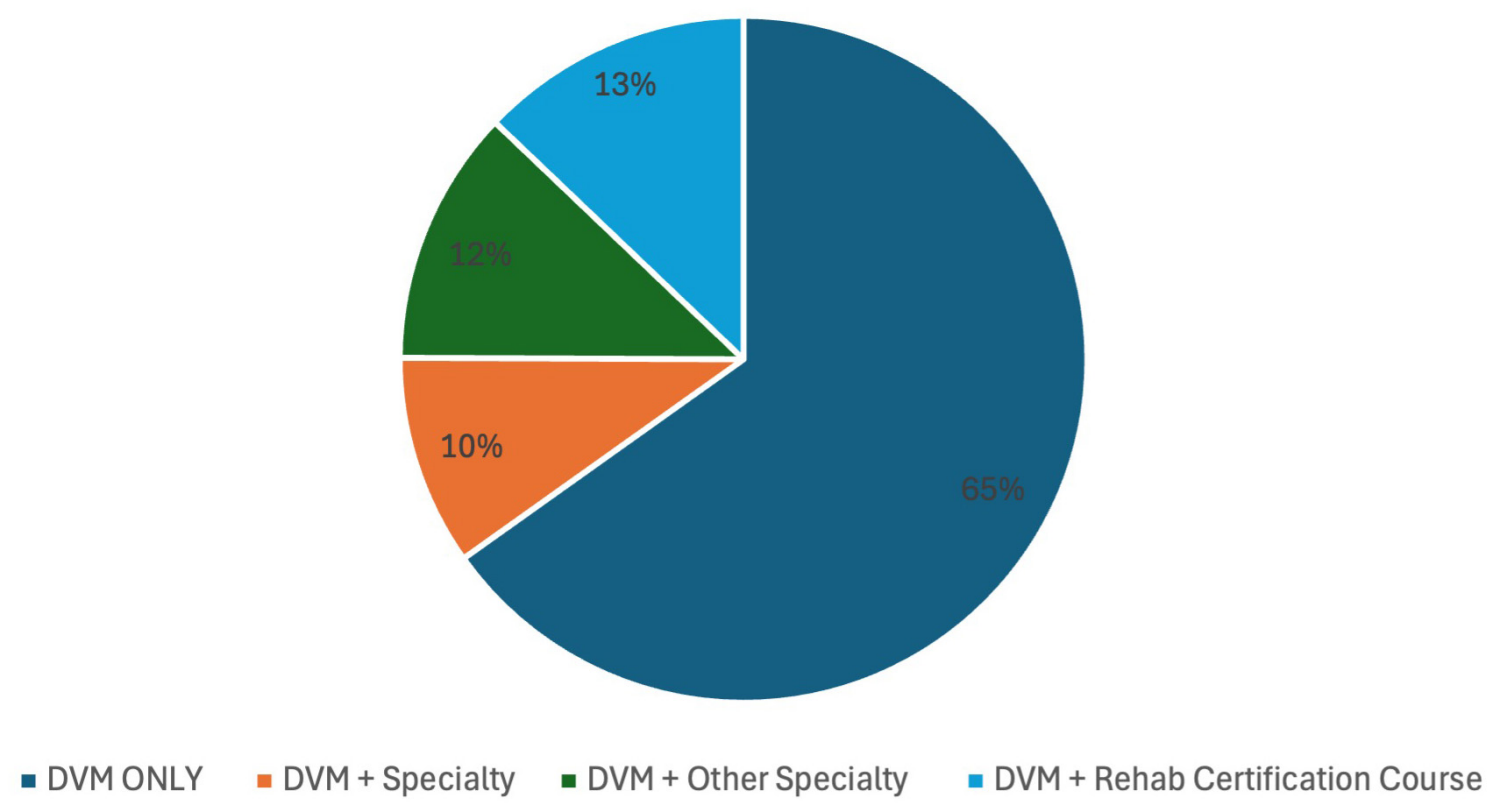
Distribution of respondents by highest level of training. DVM only = dark blue; DVM + Specialty (ACVS or ACVSMR) = orange; DVM + other specialty = green; DVM + rehab certification course = light blue.

A total of 416 veterinarians completed the survey; 373 responses were included in the final analysis, with 43 responses excluded due to incompleteness or respondent ineligibility. The majority of respondents (65.15%, *n* = 243) held a DVM or equivalent degree, without additional specialty credentials. The breakdown of the training level of respondents is shown in Figure 1. Respondents represented a broad range of experience levels. The relative years in practice of respondents are reflected in Figure 2.

### Diagnostic imaging

The use of diagnostic imaging (radiographs or CT) to confirm OA varied significantly among respondents. Overall, 53% (*n* = 203) of respondents use radiographs or CT imaging whenever possible for suspected OA cases, 36% (*n* =139) reserve imaging for cases in which clinical signs are ambiguous, and 10% (*n* = 37) rarely used imaging. Only 1% (*n* = 3) stated that they never use radiographs or CT to diagnose OA. ACVSMR and ACVS diplomates were significantly more likely to report using radiographs or CT for OA diagnosis (*p* = 0.0088 and 0.0048, respectively). Veterinarians with a rehabilitation certification also reported more frequent use of imaging (*p* = 0.0002). In contrast, DVM-only respondents were significantly less likely to use imaging for diagnostic confirmation (*p* < 0.0001). Imaging practices also differed by experience level, with veterinarians who had been in practice for fewer years being less likely to use radiographs or CT (*p* = 0.0152).

### Nonsteroidal anti-inflammatory drug usage

Prescribing frequency of NSAIDs for canine OA varied among respondents. Twenty percent of respondents (*n* = 75) prescribed NSAIDs consistently, 65% (*n* = 244) often prescribe NSAIDs, 12% (*n* = 44) sometimes prescribe NSAIDs, 3% (*n* = 12) rarely prescribe NSAIDs, and 1% (*n* = 2) never prescribe NSAIDs for OA management. ACVS diplomates prescribe NSAIDs more frequently than other respondents (*p* = 0.0052). Respondents with more years in clinical practice were significantly more likely to prescribe NSAIDs (*p* = 0.0031). Among the small subset of respondents who reported that they do not prescribe NSAIDs for OA management (*n* = 2, 1%), reasons cited included lack of long-term efficacy in pain management.

Carprofen was the most frequently prescribed NSAID (*n* = 270, 74%), followed by grapiprant (*n* = 37, 10%). There were no significant differences between respondent subgroups in the most prescribed NSAID. The most frequently reported perceived side effect of grapiprant was diarrhea (*n* = 39, 33%), followed by vomiting (*n* = 36, 31%). Other reported perceived side effects of grapiprant included elevated liver values (12%, *n* = 14), decreased appetite (*n* = 13, 11%), elevated kidney values (*n* = 11, 9%), gastrointestinal ulceration (*n* = 5, 4%), and lethargy (*n* = 5, 4%). Lack of efficacy (*n* = 6, 5%) was reported in the free-text response section regarding side effects of grapiprant. Nineteen percent (*n*=23) of grapiprant prescribers reported no perceived side effects.

The majority (n = 213, 58%) of NSAID prescribers monitored patients every 6 months, while 20% (*n* = 75) monitored patients annually. A serum chemistry panel was used by 93% (*n* = 342) of respondents, complete blood count (CBC) was used by 72% (*n* = 264), and urinalysis by 42% (*n* = 152). No significant differences were found between respondent subgroups in monitoring tests or frequency.

Among NSAID prescribers, 245 (67%) reported using NSAIDs for more than 12 months; 62 (17%) limited use to ≤ 3 months; 33 (9%) to 3–6 months; and 27 (7%) to 6–12 months. No significant differences in maximum duration were observed between respondent subgroups. Concerns for long term NSAID use included renal or hepatic damage (*n* = 59, 50%), gastrointestinal side effects (*n* = 24, 20%), lack of long-term efficacy (*n* = 10, 8%) and owner preference or cost concerns (*n* = 6, 5%). An additional 16% (*n* = 19) selected “other,” with free-text responses referencing a preference for multimodal treatment and concern for combined gastrointestinal and renal/hepatic effects. DVM-only respondents were more likely to report setting a maximum duration due to renal/hepatic concerns and perceived lack of long-term efficacy (*p* = 0.002). ACVS diplomates were more likely to cite gastrointestinal side effects as the primary reason (*p* = 0.029).

### Other pharmaceuticals

**Figure 3:**
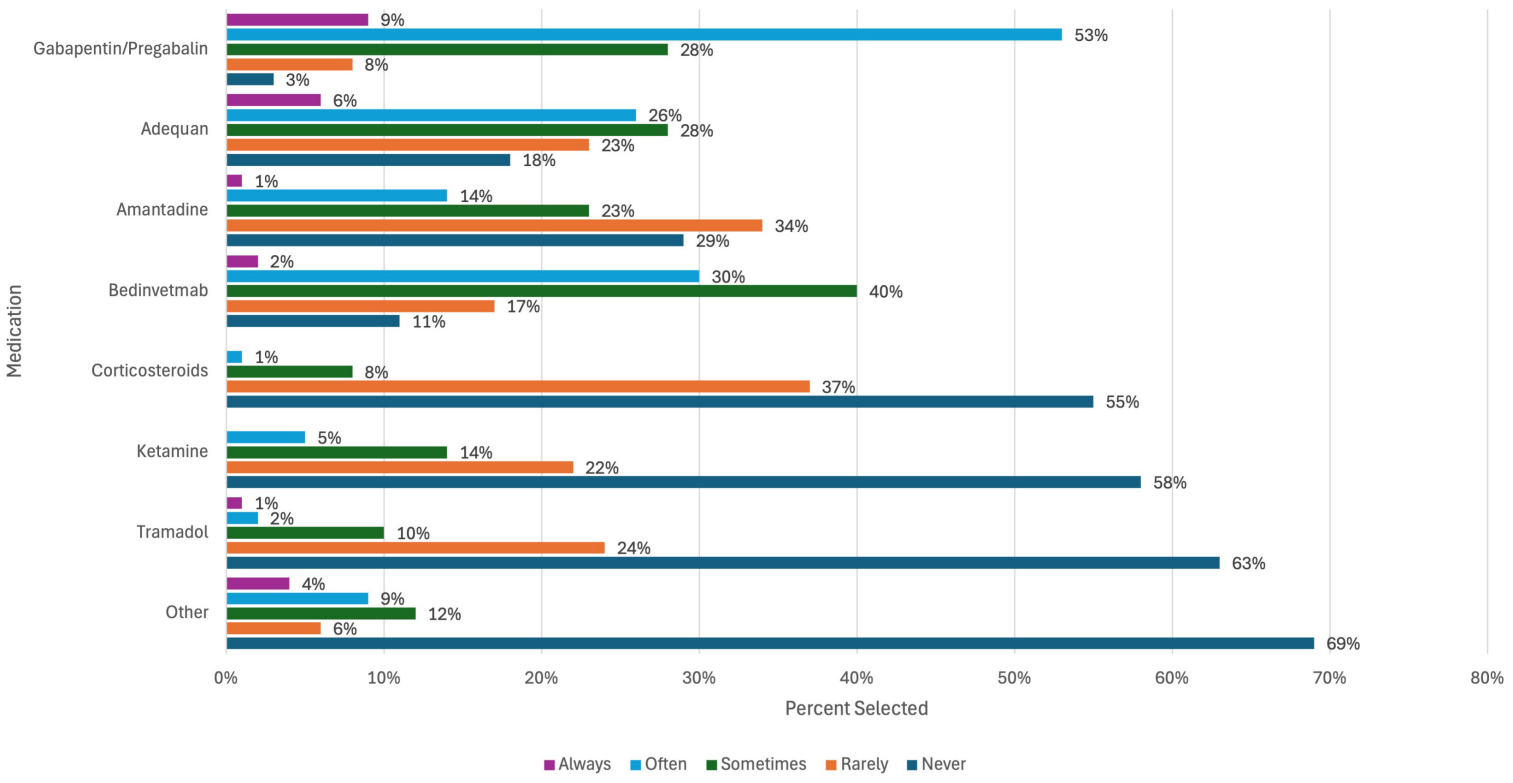
Frequency of use of non-NSAID pharmacologic treatments for the management of canine osteoarthritis among survey respondents. Purple = always; ofte*n* = light blue; sometimes = green; rarely = orange; never = dark blue. Respondents reported how frequently they prescribed each listed medication using a 5-point Likert scale (1 = Never to 5 = Always). Gabapentinoids were the most frequently prescribed with a mean Likert score of 3.56 ± 0.87, followed by bedinvetmab (2.94 ± 0.99), PSGAG (2.79 ± 1.17), amantadine (2.23 ± 1.04), “other” medications (1.73 ± 1.2), ketamine (1.66 ± 0.90), corticosteroids (1.55 ± 0.69), and tramadol (1.53 ± 0.82).

Respondents demonstrated significant variability in using non-NSAID pharmaceuticals (Figure 3). Free-text responses regarding “other” medications indicated the use of acetaminophen (*n* = 19), codeine (*n* = 9), amitriptyline (*n* = 6), pentosan polysulfate (*n* = 4), memantine (*n* = 2) methocarbamol (*n* = 2), lidocaine (*n* = 2), naltrexone (*n* = 1), nandrolone (*n* = 1), and testosterone restoration therapy (*n* = 1). ACVSMR and ACVS diplomates and rehabilitation-certified veterinarians were more likely to prescribe amantadine (*p* < 0.001, *p* = 0.0002, *p* < 0.0001, respectively). ACVSMR diplomates and rehabilitation-certified veterinarians were also more likely to prescribe ketamine (*p* < 0.0001, *p* < 0.0001), PSGAGs (*p* = 0.001, *p* < 0.0001) and more frequently selected “other” pharmacologic options not listed in the survey (*p* = 0.040, *p*=0.021). Additionally, veterinarians with more years in clinical practice were more likely to prescribe amantadine (*p* = 0.008), tramadol (*p*=0.021), and to report use of “other” medications (*p* = < 0.001).

### Anti-nerve growth factor monoclonal antibodies

**Figure 4:**
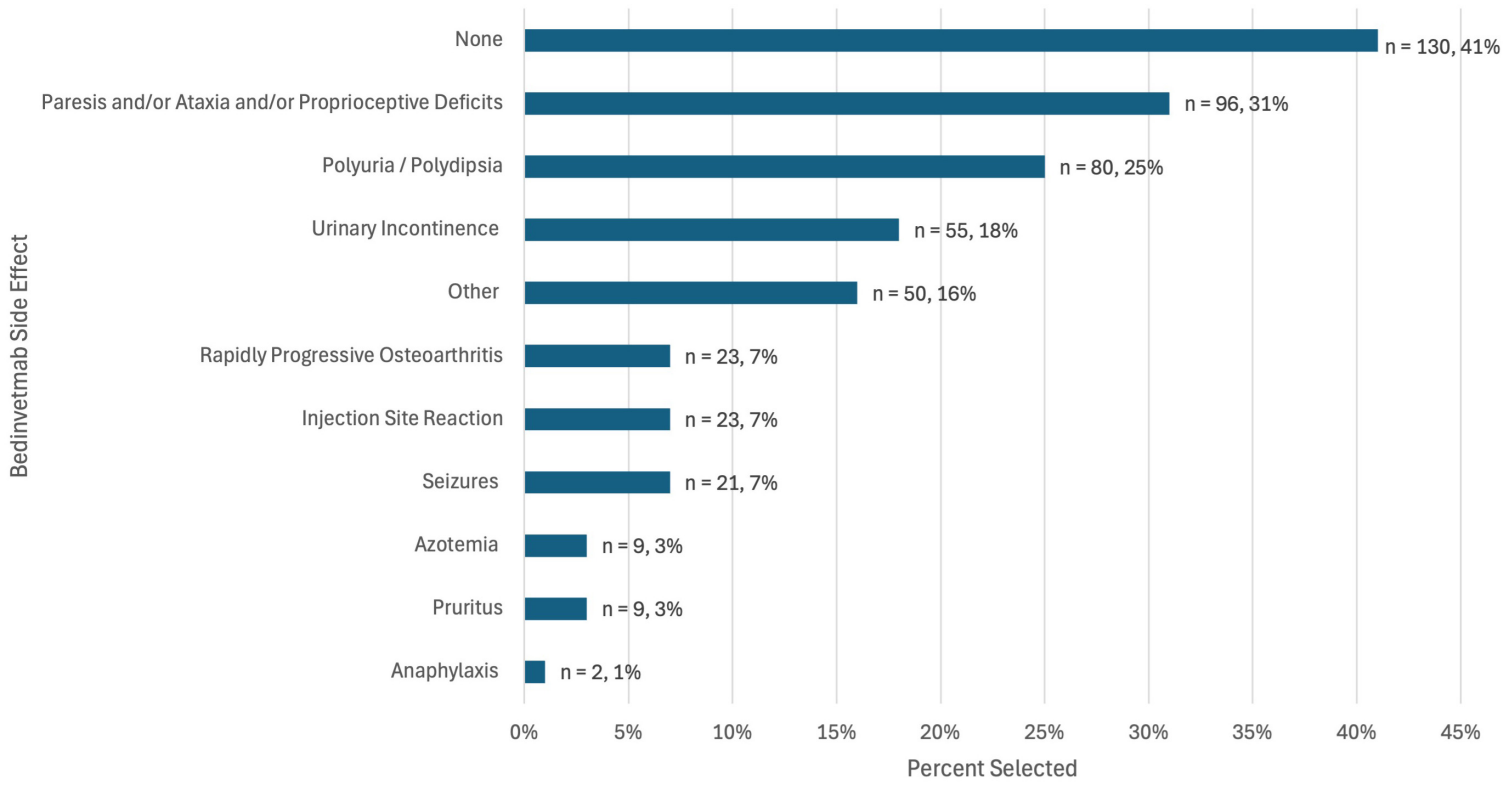
Reported side effects associated with bedinvetmab in dogs treated for osteoarthritis among survey respondents. The most reported adverse event was “None” (41%, *n* = 130). Among those who did report side effects, the most frequently reported included paresis and/or ataxia and/or proprioceptive deficits (31%, *n* = 96), polyuria/polydipsia (25%, *n* = 80), and urinary incontinence (18%, *n* = 55). Less commonly observed adverse events included rapidly progressive osteoarthritis (7%, *n* = 23), injection site reactions (7%, *n* = 23), seizures (7%, *n* = 21), azotemia (3%, *n* = 9), pruritus (3%, *n* = 9), and anaphylaxis (1%, *n* = 2). An additional 16% (*n* = 50) selected “Other”.

Bedinvetmab prescription patterns varied across respondent subgroups. Both ACVSMR and ACVS diplomates and rehabilitation-certified veterinarians prescribed bedinvetmab less frequently than other respondents (*p* < 0.001). ACVSMR diplomates, rehabilitation-certified veterinarians, and those with more years in practice were significantly less likely to use bedinvetmab long term (*p* = 0.035, *p* = 0.003, *p* = 0.004, respectively). Perceived side effects reported with the use of bedinvetmab are illustrated in Figure 4. Respondents that reported “other” side effects cited laryngeal paralysis, nonspecific pain, diarrhea, lethargy, urinary tract infections, cognitive deficits, and lack of effectiveness. ACVSMR diplomates were more likely to report adverse events associated with bedinvetmab (*p* = 0.001). There was an inverse relationship (*p* = 0.0036) between frequency of use of bedinvetmab and the number of adverse events observed. There was no statistically significant relationship between experience and incidence of adverse events of bedinvetmab.

### Nutraceuticals

**Figure 5:**
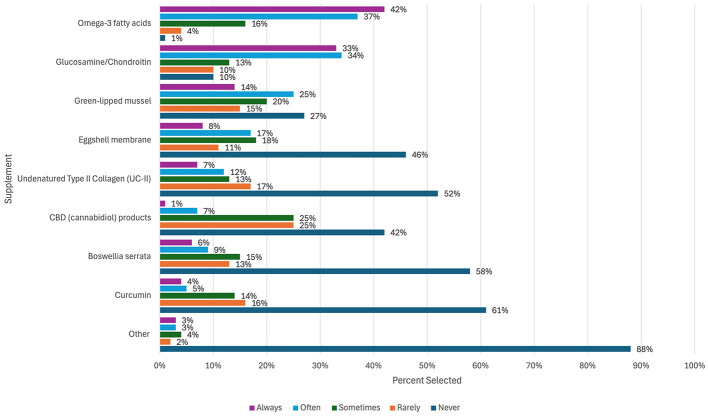
Frequency of use of nutraceuticals and joint supplements for the management of canine osteoarthritis among survey respondents. Purple = always; often = light blue; sometimes = green; rarely = orange; never = dark blue. Respondents reported how frequently they prescribed each listed medication using a 5-point Likert scale (1 = Never to 5 = Always). Omega-3 fatty acids were the most frequently prescribed (4.15 ± 0.89), followed by glucosamine/chondroitin (3.71 ± 1.29), green-lipped mussel (2.84 ± 1.42), eggshell membrane (2.29 ± 1.40), undenatured type II collagen (2.06 ± 1.33), CBD (1.99 ± 1.02), Boswellia serrata (1.93 ± 1.27), and curcumin (1.75 ± 1.11). Twelve percent of respondents indicated that they prescribe “other” supplements not listed in the survey (1.31 ± 0.93).

There was significant variation among respondents in the use of nutraceuticals for managing canine OA (Figure 5). Free responses indicated the use of Chinese herbs (*n* = 8), Traumeel^®^ (*n* = 3), Fortetropin^®^ (*n* = 5), palmitoylethanolamide (PEA, *n* = 2), Zeel^®^ (*n* = 2), methylsulfonylmethane (MSM, *n* = 2), whole eggs with shell (*n* = 1), hyaluronic acid (*n* = 3), amino acids (*n* = 1), S-Adenosyl-Methionine (SAMe, *n* = 1), 4Cyte™ (*n* = 1), dehydroepiandrosterone (DHEA, *n* = 1), ursolic acid (*n* = 1), and bromelain (*n* = 1). ACVSMR diplomates and rehabilitation-certified veterinarians were less likely to prescribe glucosamine/chondroitin compared to other respondents (*p* < 0.0001, *p* = 0.0001) and significantly more likely to prescribe several alternative supplements, including green-lipped mussel (*p* = 0.005, *p* < 0.0001), CBD (*p* < 0.001, *p* = 0.002), undenatured type II collagen (*p* < 0.0001, *p* < 0.0001), eggshell membrane (*p* = 0.012, *p* < 0.0001), Boswellia serrata (*p* = 0.001, *p* < 0.0001), curcumin (*p* = 0.001, *p* < 0.0001), and other nutraceuticals not listed in the survey (*p* = 0.017, *p* = 0.011). In contrast, ACVS diplomates were significantly less likely to prescribe CBD compared to other respondents (*p* = 0.03). Veterinarians with less clinical experience were more likely to prescribe glucosamine/chondroitin (*p* = 0.006). In contrast, those with more clinical experience were more likely to prescribe green lipped mussel (*p* < 0.001), curcumin (*p* = 0.009) and “other” joint supplements (*p* = 0.035).

### Non-pharmacological treatments

**Figure 6:**
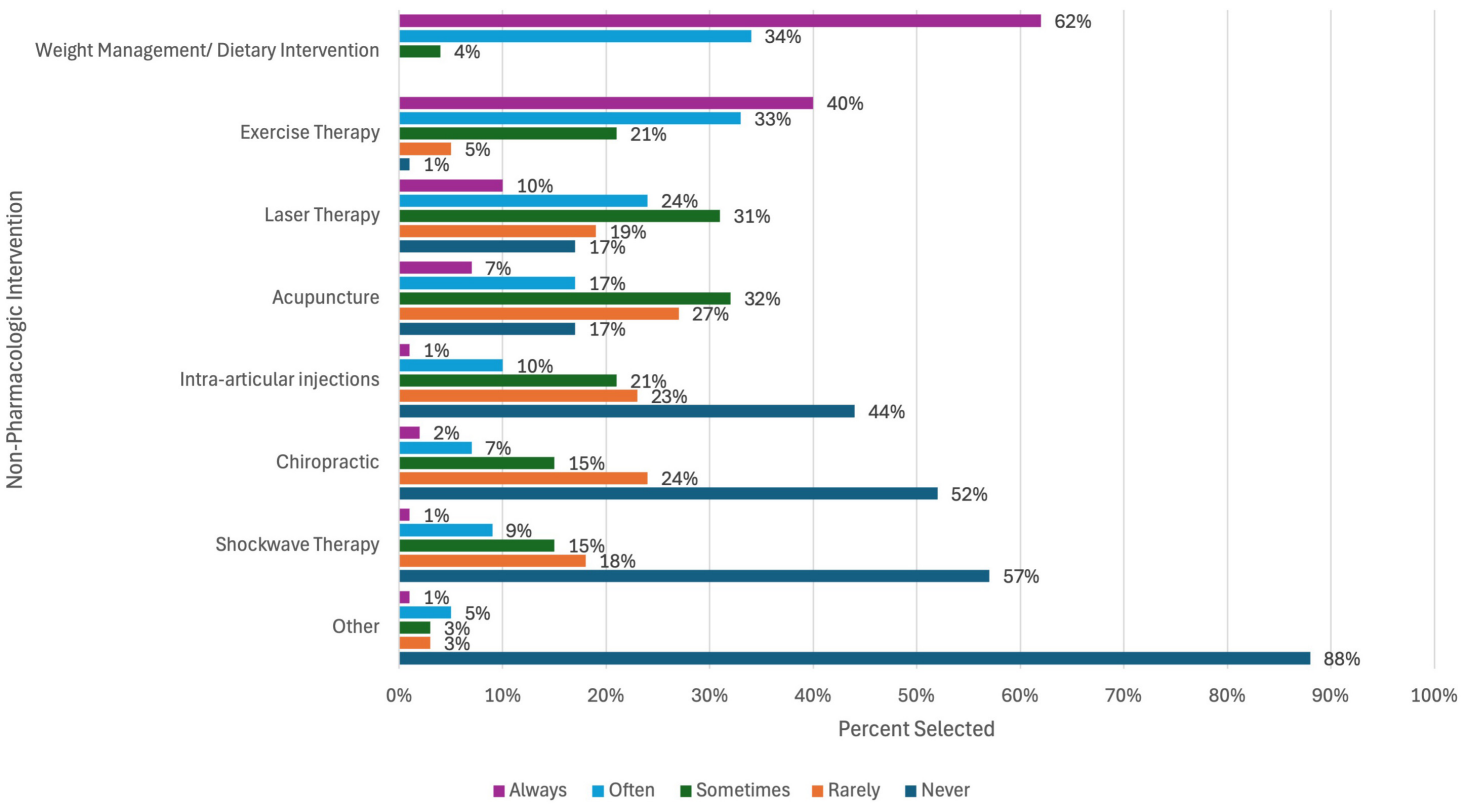
Frequency of use of non-pharmacologic therapies for the management of canine osteoarthritis among survey respondents. Purple = always; often = light blue; sometimes = green; rarely = orange; never = dark blue. Respondents reported how frequently they prescribed each listed medication using a 5-point Likert scale (1 = Never to 5 = Always). Weight management/dietary intervention was the most recommended treatment (4.57 ± 0.58), followed by exercise therapy (4.06 ± 0.94), laser therapy (2.91 ± 1.21), acupuncture (2.70 ± 1.15), intra-articular injections (2.01 ± 1.08), chiropractic (1.83 ± 1.05), and extracorporeal shockwave therapy (ESWT) (1.80 ± 1.08). Twelve percent of respondents reported the use of “other” non-pharmacological treatments that were not listed in the survey (1.29 ± 0.85).

Usage of non-pharmacological treatments for OA varied significantly among respondent subgroups (Figure 6). Respondents that selected the use of “other” non-pharmacological treatments that were not listed in the survey provided free-text responses indicating the use of pulsed electromagnetic field therapy (*n* = 11), prescription diets (*n* = 5), massage (*n* = 3), heat therapy (*n* = 3), transcutaneous nerve stimulation (*n* = 2), hyperbaric oxygen therapy (*n* = 2), total joint replacement (*n* = 1), and interfascial blocks (*n* = 1). ACVSMR diplomates and rehabilitation-certified veterinarians were significantly more likely to recommend exercise therapy (*p* < 0.001), acupuncture (*p* < 0.001), chiropractic (*p* = 0.001, *p* < 0.0001), ESWT (*p* < 0.0001), IA injections (*p* < 0.0001), and laser therapy (*p* < 0.0001). ACVSMR diplomates were also more likely to prescribe “other” therapies (*p* = 0.010). ACVS diplomates reported more frequent use of ESWT (*p* = 0.007) and IA injections (*p* = 0.0001). Veterinarians with more years in practice were more likely to utilize exercise therapy (*p* = 0.001), chiropractic (*p* < 0.001), laser therapy (*p* = 0.016), IA injections (*p* = 0.002), and “other” therapies (*p* = 0.001).

DVM only = dark blue; DVM + Specialty (ACVS or ACVSMR) = orange; DVM + other specialty = green; DVM + rehab certification course = light blue.

## Discussion

### Overview

Despite the high prevalence of canine OA, limited literature exists describing how treatment strategies vary among veterinarians. The results of this study revealed considerable variation in multimodal approaches to OA influenced by both specialty training and years of clinical experience. Thus, our hypothesis that OA management practices would differ by training and experience was supported.

Consensus frameworks aim to standardize the diagnosis and treatment of OA. The Canine OsteoArthritis Staging Tool (COAST) Development Group proposed guidelines for the assessment and monitoring of canine OA. The COAST Stage excluding radiography (COASTeR stage) tool has also been developed to guide treatment and management choices, encompassing a staged, multimodal approach to stage-specific management of canine OA (16). Although guidelines exist to standardize diagnosis and care, findings from this study suggest that significant variability is common in practice likely secondary to a variety of factors including experience level, training, and accessibility. These factors are further explored in the sections below.

### Diagnostic imaging

Most respondents reported using diagnostic imaging whenever possible in suspected OA cases. This is consistent with recommendations from the COAST guidelines in which diagnosis by radiographs or CT are considered standard of care (16, 17). Notably, ACVSMR and ACVS diplomates, as well as DVMs with rehabilitation certifications, were significantly more likely to use diagnostic imaging “whenever possible for all suspected OA cases.” This is likely reflective of the greater emphasis on diagnostics and comprehensive treatment plans in advanced training programs. Although clients seeking specialty care may also be more willing to pursue imaging than those in general practice, the authors worded this item to minimize population bias. Additionally, veterinarians with fewer years of experience were less likely to utilize diagnostic imaging, which may reflect a greater reliance on history and physical examination findings, reduced awareness of differential diagnoses, or reduced confidence with interpreting imaging. These patterns highlight the need for consistency in diagnostic protocols and training in OA staging.

### Nonsteroidal anti-inflammatory drug usage

NSAIDs remain a mainstay of canine OA treatment. Most respondents reported prescribing NSAIDs either consistently or frequently, with the most frequently prescribed NSAID being carprofen, followed by grapiprant. These findings align with prior studies supporting carprofen's safety and efficacy (18) and growing evidence for grapiprant in multimodal protocols (19). Side effects reported for grapiprant in this study included diarrhea and vomiting, consistent with prior safety studies (20, 21). No side effects were reported by 19% (*n* = 23) of grapiprant prescribers. However, elevated liver values, azotemia, and GI ulceration were also observed, which have not been previously reported in the literature. Given the survey nature of this study, it is unclear if these adverse events were truly caused by grapiprant. ACVS diplomates and more experienced clinicians were more likely to report frequent NSAID use. ACVS diplomates may use more NSAIDS due to treating a high volume of post-surgical OA cases. Both ACVS diplomates and more experienced clinicians may also be more aware of evidence supporting efficacy of NSAIDs.

Most clinicians monitored NSAID therapy by performing laboratory monitoring every 6 months, typically using serum chemistry panels. This is consistent with guidelines from the American Animal Hospital Association (22) and previous research showing high concern among veterinarians and owners regarding NSAID risks and reliance on routine bloodwork (23) to ensure safety. Most respondents supported long-term NSAID use (>12 months). However, renal and hepatic concerns were more frequently cited by DVM-only respondents, while ACVS diplomates more often cited concerns about gastrointestinal effects. Existing literature supports the long-term safety of NSAIDs in OA when appropriately monitored (24). To date, no published studies have demonstrated an increased incidence of gastrointestinal, hepatic, or renal adverse events specifically associated with extended NSAID therapy in canine OA patients.

### Other pharmaceuticals

Beyond NSAIDs, gabapentinoids, bedinvetmab, and PSGAG were the most frequently prescribed pharmaceuticals. Gabapentin has been found to be well-tolerated, with a large dosing range (25). Despite the lack of literature supporting the efficacy of gabapentinoids for the treatment of OA related pain, these were the most prescribed pharmaceuticals aside from NSAIDs. There have been several double-blinded clinical trials supporting the efficacy of bedinvetmab (21, 26–28). There is currently only moderate evidence for the efficacy of PSGAG, and further studies are needed (18). In the present study, free-text responses also revealed a variety of additional prescribed pharmacologics highlighting the individualized approaches veterinarians take when managing OA pain. ACVSMR diplomates and rehabilitation-certified veterinarians reported greater use of ketamine and “other” medications which may reflect the higher severity of OA cases that present to rehabilitation practices. Veterinarians with more years of practice were more likely to report prescribing amantadine and tramadol, as well as other medications not included in the survey. This may reflect a broader familiarity with adjunctive therapies developed over time or greater clinical confidence in employing less conventional options. These findings support previous calls for larger-scale studies to better define the efficacy of emerging pharmaceuticals (29).

### Anti-nerve growth factor monoclonal antibodies

Bedinvetmab (Librela^TM^), a canine anti-nerve growth factor monoclonal antibody, has been proposed as a first-line medication for chronic pain management of OA (30) and several robust clinical trials have demonstrated bedinvetmab's efficacy in reducing OA-associated pain (26, 27). In this study, ACVS and ACVSMR diplomates, and rehabilitation-certified veterinarians prescribed bedinvetmab less frequently which may reflect recent concerns about associated adverse events (31–34). In addition, ACVSMR diplomates, rehabilitation-certified veterinarians, and veterinarians with greater clinical experience were less likely to use bedinvetmab long term. These differences between groups may be influenced by external information sources. Exposure to social media may also influence adverse event reporting patterns (35). ACVS and ACVSMR diplomates and rehabilitation-certified veterinarians may be more likely to participate in online communities related to OA therapies, which could increase awareness of potential adverse events.

Reported adverse events were consistent with previous publications, including polyuria/polydipsia and urinary incontinence, and ataxia (33). The incidence of these adverse events is confounded by the fact that bedinvetmab is most commonly administered to older dogs (36) which have a high baseline incidence of neurological disease (37). From the existing literature alone, it cannot be determined whether dogs with reported neurological side effects had preexisting neurological disease. Recent studies have raised concerns about potential links between bedinvetmab and musculoskeletal conditions such as ligament injuries and polyarthritis (34). In this study, 7% of bedinvetmab prescribers reported rapidly progressive osteoarthritis (RPOA) as an adverse event. Whether there is a causal link between bedinvetmab and RPOA remains controversial (33, 34). Further investigation into suspected RPOA cases is warranted to help distinguish true rapidly progressive disease from other disorders such as IMPA or natural OA progression.

Although the survey did not explicitly inquire about the incidence of lack of efficacy in response to bedinvetmab, approximately 1% (4 out of 365) of respondents who prescribed this medication reported lack of efficacy. This is higher than what has been previously reported by a prior pharmacovigilance study in which there were 1.7 reports of lack of efficacy out of 10,000 animals (33). However, these differences should be interpreted cautiously as pharmacovigilance reporting tends to be more biased toward severe adverse events (38) which may lead to underreporting lack of efficacy.

### Nutraceuticals

Omega-3 fatty acids were the most reported nutraceutical used among respondents, consistent with the high level of evidence supporting their efficacy (39, 40). A recent meta-analysis of nutraceuticals for canine OA concluded that omega-3 fatty acid enriched diets, omega-3 fatty acid supplements, and CBD nutraceuticals are effective in alleviating OA-related pain in dogs (40). While some studies suggest that glucosamine/chondroitin may have a long-term protective effect on cartilage (41), the meta-analysis of canine clinical trials showed no significant effect of glucosamine/chondroitin products in managing OA-related clinical signs (40). Despite this, glucosamine/chondroitin was the second most frequently prescribed nutraceutical by respondents of this study. However, it was prescribed less frequently by ACVSMR diplomates and rehabilitation-certified veterinarians, possibly suggesting greater awareness of emerging research questioning its efficacy (40, 42). Veterinarians with less clinical experience, however, were more likely to prescribe glucosamine/chondroitin, suggesting opportunities for continuing education. Additionally, ACVSMR diplomates and rehabilitation-certified veterinarians were more likely to prescribe alternative supplements, such as CBD, while ACVS diplomates were significantly less likely to prescribe these. Interestingly, CBD and green-lipped mussel were prescribed less frequently than glucosamine/chondroitin, despite available evidence supporting their safety and efficacy for managing canine OA-related pain (43–46).

### Non-pharmacological treatments

Weight management was the most prescribed non-pharmacological treatment among respondents in this study, consistent with the strong evidence for its role in improving lameness and delaying or preventing OA progression (8, 47). This finding aligns with the COAST development group's consensus guidelines, which emphasize weight optimization as a core intervention and support rehabilitation therapies (including exercise and ESWT), acupuncture, and intra-articular injections as part of a multimodal, stage-appropriate OA management strategy (53). In this study, ACVSMR, ACVS diplomates, and rehabilitation-certified veterinarians were significantly more likely to recommend several interventions, including ESWT, and IA injections. Veterinarians with more years in practice were more likely to utilize exercise therapy, laser, chiropractic, and IA injections. These trends may reflect both greater access to resources and training, as well as more experience with multimodal OA management plans.

## Limitations

There were several limitations of this study. First, the survey design introduces the potential for selection bias, as participation was voluntary and may have attracted respondents with a particular interest in OA management. Additionally, the convenience sampling strategy likely caused an uneven distribution of subgroup sizes, such as ACVS and ACVSMR diplomates, which limits the strength of group comparisons. Described variations in OA management strategies between groups could be due to a variety of factors, including differences in the types of cases and/or client goals presented to veterinarians with different expertise, and should not be interpreted as solely due to variations in training. This survey relied on self-reported data, which is susceptible to recall bias. Without linkage to patient outcomes, it is not possible to confirm whether reported practices or perceived side effects align with adverse event data. A notable limitation of the survey design was the use of free text “other” response fields within multiple sections, specifically those regarding frequency of use of non-NSAID pharmaceuticals, nutraceuticals, and non-pharmacologic treatments. While these fields were included to allow respondents to report additional treatments not listed in the structured survey options, some respondents entered treatments that were already included in other sections. Additionally, although respondents were able to use a Likert sale to indicate frequency of use for the “other” textbox, many free text responses contained multiple entries, which prevented accurate attribution of the Likert rating to any one specific treatment, rendering those results unsuitable for statistical analysis. Another limitation of this study was that there were no questions pertaining to usage of outcome measures to assess OA treatment response. Due to the cross-sectional nature of the survey, it was not possible to gauge how respondents determine treatment success or adjust treatment protocols over time.

## Conclusion

This survey highlights the variation in multimodal approaches in the management of canine OA among veterinarians. While NSAIDs and weight management remain foundational, this study demonstrates a spectrum of adjunctive strategies, including alternative pharmaceuticals, nutraceuticals, and non-pharmacologic therapies. Significant variation in prescribing patterns was observed across subgroups of veterinarians, suggesting that training background and clinical experience affect decision-making. Reported adverse events associated with recently approved medications for OA, bedinvetmab, and grapiprant, underscore the need for pharmacovigilance and further clinical research investigating their safety, especially in diverse patient populations.

## Data Availability

The raw data supporting the conclusions of this article will be made available by the authors, without undue reservation.
