# Supplementary material for: Variations in the management of canine osteoarthritis: a cross-sectional survey of veterinary practices
Source: Front Vet Sci. 2026 May 29;13:1814641. doi: 10.3389/fvets.2026.1814641 (PMC13259648; doi:10.3389/fvets.2026.1814641)
Supplement: Supplementary file 1 [file Supplementary_file_1.pdf]

Supplementary Material S1. Survey Instrument

Canine Osteoarthritis Management Survey

Formatting note. The survey is presented in a streamlined format for publication as supplementary material. Qualtrics interface text, administrative controls, timestamps, page URLs, and duplicate builder artifacts were removed. The numbering in the left column corresponds to Qualtrics export variable IDs from the spreadsheet header rows. Display logic is retained where applicable.

Frequency scale used in matrix questions: Never; Rarely; Sometimes; Often; Always. For NSAID use, the administered anchors were: Never; Rarely (less than 10% of OA cases); Sometimes (10-50% of OA cases); Often (50-90% of OA cases); Always (more than 90% of OA cases).

Section 1. Respondent Demographics

Qualtrics ID(s)	Survey question	Response options / format
QID1	How many years have you been in veterinary practice?	0-5 years; 6-10 years; 11-20 years; More than 20 years.
QID2_1-QID2_8; QID2_7_TEXT	What degrees, certifications, or diplomas do you possess? Select all that apply.	DVM or equivalent (QID2_1); ACVSMR and/or ECVSMR, or other VSMR board certification (QID2_2); ACVS and/or ECVS, or other surgical board certification (QID2_3); Rehabilitation certification, CCRT/CCRP/CCRV or equivalent (QID2_6); ACVIM (QID2_8); Other (QID2_7); Other text (QID2_7_TEXT).

Section 2. Diagnostic Practices for OA

Qualtrics ID(s)	Survey question	Response options / format
QID3	When do you use radiographs or CT to confirm a diagnosis of osteoarthritis (OA) in dogs?	Whenever possible for all suspected OA cases; Only if clinical symptoms are ambiguous; Rarely, because clinical symptoms are sufficient for diagnosis; Never, I do not use radiographs for OA diagnosis.

Section 3. Pharmacological Management of OA: NSAIDs

Qualtrics ID(s)	Survey question	Response options / format
QID4	How frequently do you prescribe NSAIDs for OA management in dogs?	Never; Rarely (less than 10% of OA cases); Sometimes (10-50% of OA cases); Often (50-90% of OA cases); Always (more than 90% of OA cases).
QID16; QID16_5_TEXT	What is your primary reason for not prescribing NSAIDs for management of OA in dogs?	Displayed if QID4 = Never. Options: Concern about gastrointestinal side effects (vomiting, diarrhea, ulcers); Concern about renal or hepatic damage; Lack of long-term efficacy in pain management; Owner preference or cost concerns; Other (please specify; QID16_5_TEXT).
QID6; QID6_6_TEXT	Which NSAID do you prescribe most frequently for canine osteoarthritis?	Carprofen (Rimadyl); Meloxicam (Metacam); Firocoxib (Previcox); Deracoxib (Deramaxx); Grapiprant (Galliprant); Other (please specify; QID6_6_TEXT).
QID7	How frequently do you monitor dogs on long-term NSAIDs for OA?	Monthly; Every 3 months; Every 6 months; Annually; Only if clinical signs of adverse effects appear.
QID8_1-QID8_6; QID8_5_TEXT	Which tests do you typically run to monitor dogs on long-term NSAIDs for OA? Select all that apply.	Complete Blood Count/CBC (QID8_1); Serum Chemistry Panel/Chem (QID8_2); Urinalysis/UA (QID8_6); Other (QID8_5); Other text (QID8_5_TEXT).
QID9	When treating OA in dogs, what is the maximum duration you typically recommend for the continuous use of NSAIDs?	Less than 3 months; 3-6 months; 6-12 months; More than 12 months (long-term use without a set maximum).

Qualtrics ID(s)	Survey question	Response options / format
QID10; QID10_6_TEXT	What is your primary reason for setting this maximum duration for NSAID use?	Displayed unless QID9 = More than 12 months. Options: Concern about gastrointestinal side effects (vomiting, diarrhea, ulcers); Concern about renal or hepatic damage; Lack of long-term efficacy in pain management; Owner preference or cost concerns; Other (please specify; QID10_6_TEXT).
QID20_1-QID20_10; QID20_9_TEXT	What side effects, if any, have you observed in dogs treated with Galliprant? Select all that apply.	N/A - I do not prescribe Galliprant (QID20_10); None (QID20_1); Vomiting (QID20_2); Diarrhea (QID20_3); Decreased appetite (QID20_4); Lethargy (QID20_5); GI ulceration (QID20_6); Elevated liver values (QID20_7); Elevated kidney values (QID20_8); Other (QID20_9); Other text (QID20_9_TEXT).

Section 4. Pharmacological Management of OA: Non-NSAID Medications

QID11. How often do you prescribe the following medications for OA management in dogs?

Response scale for each row: Never; Rarely; Sometimes; Often; Always.

Qualtrics ID	Matrix row / item	Response format
QID11_1	Gabapentin/Pregabalin	Frequency scale
QID11_2	Adequan	Frequency scale
QID11_3	Amantadine	Frequency scale
QID11_4	Tramadol	Frequency scale
QID11_5	Corticosteroids	Frequency scale
QID11_6	Librela (bedinvetmab)	Frequency scale
QID11_7	Ketamine	Frequency scale
QID11_8; QID11_8_TEXT	Other (select Never if not applicable)	Frequency scale; free-text field for Other

Section 5. Pharmacological Management of OA: Bedinvetmab Details

Qualtrics ID(s)	Survey question	Response options / format
QID12_1-QID12_11; QID12_7_TEXT	If you have prescribed Librela, what side effects have you observed? Select all that apply.	Displayed if QID11_6 = Rarely, Sometimes, Often, or Always. Options: Polyuria/polydipsia (QID12_1); Injection site reaction (QID12_2); Urinary incontinence (QID12_9); Paresis and/or ataxia and/or proprioceptive deficits (QID12_3); Seizures (QID12_11); Azotemia (QID12_4); Pruritus (QID12_10); Anaphylaxis (QID12_5); Rapidly progressive osteoarthritis (QID12_6); Other (QID12_7); Other text (QID12_7_TEXT); None (QID12_8).
QID19; QID19_7_TEXT	For dogs treated with Librela, after how many months, if at all, do you typically consider tapering or discontinuing its usage?	Displayed if QID11_6 = Rarely, Sometimes, Often, or Always. Options: Never; 2-3 months; 4-6 months; 7-9 months; 10-12 months; Over 12 months; Other (please specify; QID19_7_TEXT).

Section 6. Nutraceuticals and Supplements

QID13. How often do you prescribe each of the following joint supplements for OA management?

Response scale for each row: Never; Rarely; Sometimes; Often; Always.

Qualtrics ID	Matrix row / item	Response format
QID13_1	Glucosamine/Chondroitin	Frequency scale
QID13_2	Omega-3 fatty acids	Frequency scale
QID13_3	Green-lipped mussel	Frequency scale
QID13_4	CBD (cannabidiol) products	Frequency scale
QID13_5	Undenatured Type II Collagen (UC-II)	Frequency scale
QID13_6	Eggshell membrane	Frequency scale
QID13_7	Boswellia serrata	Frequency scale
QID13_8	Curcumin	Frequency scale

Qualtrics ID	Matrix row / item	Response format
QID13_9; QID13_9_TEXT	Other (select Never if not applicable)	Frequency scale; free-text field for Other

Section 7. Non-pharmacological Management of OA

QID14. How often do you prescribe the following non-pharmacological treatments for managing OA in dogs?

Response scale for each row: Never; Rarely; Sometimes; Often; Always.

Qualtrics ID	Matrix row / item	Response format
QID14_1	Weight management / dietary intervention	Frequency scale
QID14_2	Exercise therapy	Frequency scale
QID14_3	Acupuncture	Frequency scale
QID14_4	Chiropractic	Frequency scale
QID14_5	Laser therapy	Frequency scale
QID14_6	Shockwave therapy	Frequency scale
QID14_7	Intra-articular injections (e.g., corticosteroids, hyaluronic acid, hydrogels, platelet-rich plasma)	Frequency scale
QID14_8; QID14_8_TEXT	Other (select Never if not applicable)	Frequency scale; free-text field for Other

End of survey: We thank you for your time spent taking this survey. Your response has been recorded.
